# Materiobiology in the omics era

**DOI:** 10.1016/j.mtbio.2025.102539

**Published:** 2025-11-10

**Authors:** Peiran Song, Yuezhou Wu, Chen Zhang, Fengjin Zhou, Long Bai, Jiacan Su

**Affiliations:** a MedEng-X Institutes, Shanghai University, Shanghai, 200444, China; b Organoid Research Center, Institute of Translational Medicine, Shanghai University, Shanghai, 200444, China; c National Center for Translational Medicine (Shanghai) SHU Branch, Shanghai University, Shanghai 200444, China; d Department of Orthopedics, Xinhua Hospital Affiliated to Shanghai Jiao Tong University School of Medicine, Shanghai, 200092, China; e Wenzhou Institute of Shanghai University, Wenzhou 325000, China; f Department of Orthopedics, Honghui Hospital, Xi'an Jiao Tong University, Xi'an, 710000, China

**Keywords:** Materiobiology, Omics technologies, Biomaterial interactions, Bone organoids, Regenerative medicine

## Abstract

Omics technologies can uncover the complex regulatory mechanisms of living systems at multiple levels, including genes, transcripts, proteins, metabolites and immune networks. They also provide an integrative framework to study how cells interact with their environment. In materiobiology, cellular responses to materials involve diverse processes. This review summarizes the applications of single-cell transcriptomics, transcriptomics, RNomics, genomics, proteomics, metabolomics, lipidomics, glycomics and immunomics in materiobiology. We highlight how these approaches reveal cell heterogeneity, transcriptional and epigenetic regulation, genetic determinants, protein and metabolic pathways, lipid and glycan remodeling and immune networks in the context of biomaterial interactions. In addition, we discuss how multi-omics strategies support the construction of bone organoids, which serve as physiologically relevant models to investigate bone development, disease mechanisms and material-driven repair. Together, these advances provide a theoretical foundation and methodology for the rational design of next-generation biomaterials with improved functionality and precision in regenerative medicine. This review outlines how multi-omics technologies drive materiobiology, bridging molecular insights with material innovation for precision and regenerative medicine.

## Graphical abstract

**Figure:**
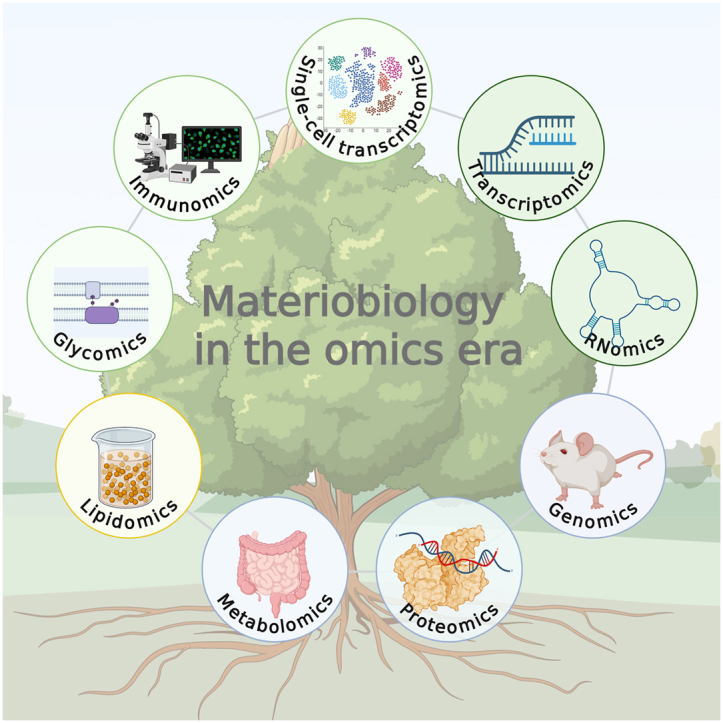

## 1 Introduction

Materiobiology is an emerging interdisciplinary discipline that mainly studies how the physical and chemical properties of biomaterials affect the biological functions of cells, tissues and organs. Traditional biological research focuses more on biochemical factors such as growth factors and cytokines. However, many studies show that stiffness, topography, surface chemistry and degradation dynamics of materials can also strongly affect cell adhesion, migration, proliferation, differentiation and stem cell renewal [1]. This idea led to the concept of materiobiology, which aims to explore the dynamic interactions between cells and materials and use them as a key entry point for tissue repair and regeneration. The integration of material science and life science has deepened. Clinical applications, such as artificial joints, bone scaffolds, vascular stents, and nerve conduits, have proven that materials are not only passive supports but also active regulators of biological behavior [2]. Yet, cell - material interactions are highly complex, driven by mechanical signaling, metabolic reprogramming and immune microenvironments. Experience-based methods are not sufficient to uncover this complexity and limit the efficiency of developing next-generation biomaterials [3].

With the rapid progress of omics technologies, researchers now use genomics, transcriptomics, proteomics, metabolomics, lipidomics, glycomics and immunomics to study cellular responses from molecular to system levels. Single-cell transcriptomics reveal heterogeneity and lineage trajectories of cells in material environments. Transcriptomics and RNomics uncover the roles of transcriptional regulation and noncoding RNAs. Genomics explains how genetic background affects bone formation, immune rejection and regeneration potential. Proteomics and metabolomics show how signaling networks and energy metabolism are reshaped by materials. The study of lipidomics and glycomics found that changes in lipids and sugar chains in cell membranes play an important role in regulating immune responses. Immunomics helps us understand the reaction process of innate immunity and adaptive immunity from an overall perspective [[4], [5], [6]].

**Fig. 1:**
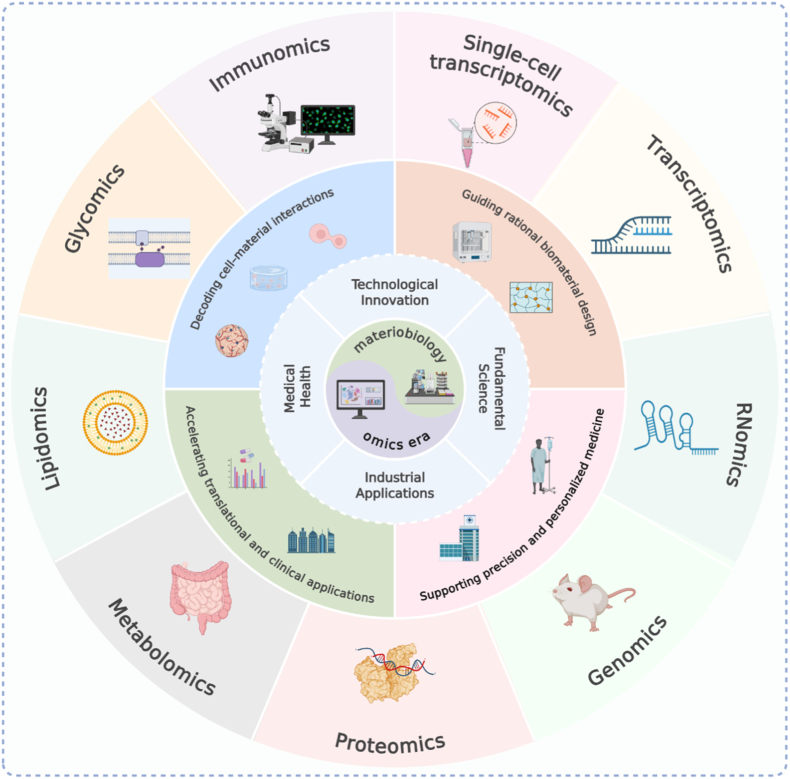
Materiobiology in the omics era.

Materiobiology is gradually shifting from empirical observation-based research to data and mechanism-based research. By integrating multi-layer tomic data, researchers can more clearly describe the process of how cells perceive and respond to different material environments. These discoveries enable people to choose suitable materials according to the specific needs of patients and design advanced biomaterials that conform to the concept of precision medicine. This transformation not only promotes the development of tissue engineering and regenerative medicine, but also lays a more solid foundation for the clinical application and industrialization of biomaterials [[7], [8], [9]] (Fig. 1). For example, Autefage and others used whole genome chip analysis to study the role of strontium. It was found that after the introduction of strontium, the expression of osteogenic-related genes changed and the synthesis pathways of methyl hydroxyvalproate and steroids/sterols were activated. These changes are accompanied by an increase in intracellular cholesterol and lipid raft, as well as an increase in the level of myoglobin light chain phosphorylation, indicating that the cell membrane structure and signal conduction process have been significantly reshaped. This indicates that the biological function of strontium is far from directly promoting osteogenesis. It may also affect the final fate of cells by regulating lipid raft dynamics and cytoskeleton signals. These findings provide clues for targeting specific molecular targets and establish a mechanism framework that can be used to guide the design of strontium based biomaterials, so as to optimize bone regeneration strategies [10]. At the same time, Groen et al. combined transcriptomics, principal component analysis and pathway enrichment analysis, and found that the material has a significant impact on many signaling pathways related to protein synthesis and energy metabolism, such as eIF2 signaling pathway, mTOR pathway, oxidative phosphorylation and glycolysation process. In addition, they also observed significant changes in the genes associated with cytoskeleton remodeling, integtin signaling and cell adhesion. These results show that the physical and chemical properties of the surface of the material will affect the metabolic state, proliferation activity and adhesion ability of osteoblasts. This study shows that transcriptomics helps to reveal the molecular mechanism of materials regulating cell function, and provides a scientific basis for optimizing the surface properties and structural design of biomaterials [11].

The results of tonomic research can help determine the key genes, signaling pathways and microenvironmental factors that affect tissue formation, thus providing direct guidance for the design and construction of organ-likes. Organ-like technology has established a new connection between materials science and biological function, enabling researchers to understand how biomaterials guide tissue formation in a way closer to the physiological state [12]. Organoids are self-organizing three-dimensional structures derived from adult or pluripotent stem cells that recapitulate the cellular architecture and functional complexity of native tissues [13]. In the field of bone research, bone organoids have become an innovative platform in recent years, which can simulate the process of ossification, angiogenesis and matrix remodeling under controllable conditions [14]. For example, Wang et al. fabricated bioprinted bone organoids using a bone matrix-inspired hybrid bioink composed of GelMA, AlgMA and hydroxyapatite. The constructs exhibited spontaneous mineralization and supported multicellular differentiation during long-term culture. Transcriptomic profiling further revealed activation of osteogenic and angiogenic gene networks, providing molecular evidence for how biomaterial composition and structure regulate bone tissue formation [15].

Materiobiology is an interdisciplinary field that connects materials science and life science, and it is now entering a new stage driven by multi-omics research. This review summarizes the applications of single-cell transcriptomics, transcriptomics, RNomics, genomics, proteomics, metabolomics, lipidomics, glycomics and immunomics in materiobiology. By analyzing molecular mechanisms, researchers can better understand how biomaterials guide cell fate, reshape the immune microenvironment and promote tissue repair. Omics technologies provide systematic data support for the rational design and functional optimization of biomaterials and also offer new ways to predict their *in vivo* behavior and reveal the complex interactions between materials, cells and tissues. In the future, with the integration of multi-omics data with artificial intelligence (AI) and big data analysis, materiobiology is expected to move from experience-driven to mechanism-driven, and from single-function to multi-functional integration. This progress will further promote the development of personalized medicine and regenerative medicine. This review highlights how multi-omics integration accelerates materiobiology, connecting molecular understanding to the rational design of advanced biomaterials.

## 2 Materiobiology and its development

Materiobiology connects materials science, biology and medicine, focusing not only on biocompatibility, but also on how materials actively regulate cell fate, immune response and tissue regeneration. The current research mainly focuses on several aspects, including mechanical biology and signal transduction, immune and metabolic regulation, stem cell differentiation and tissue remodeling. For example, the stiffness, morphology and degradation rate of the material can regulate mechanically sensitive signaling pathways such as YAP/TAZ and mTOR, thus affecting the differentiation of bone formation or angiogenesis. At the same time, the interaction between materials and immune cells determines the inflammatory response and healing results, which closely links material biology with bone immunology. More broadly, integrated system biology and omics analysis can construct a global map of how materials reshape cell heterogeneity, transcriptional activity and metabolic state, and provide a mechanism basis for personalized biological material design [1]. Early research mainly examines only one variable at a time, such as surface chemical properties or stiffness, and conducts a gentative evaluation of cell adhesion or proliferation. Although these methods provide useful information, they are limited. Modern material biology adopts high-throughpult screening, microarray platform and micro-flow control system, which can test hundreds of material compositions and structures in a controlled microenvironment at the same time. High connotation imaging, computational modeling and machine learning further enhance the ability to quantify complex cell behavior and predict material properties [3,16]. At the same time, the integration of genomics, transcriptomics, proteomics and metabolomics has given people a molecular-level understanding of the interaction between materials and cells. Combining these group data sets with AI and big data analysis, key genes, pathways and molecular networks involved in the material-guided regeneration process can be identified. Together, these advances have transformed materiobiology from a descriptive discipline to a mechanical and predictive science. The integration of group technology, AI and digital manufacturing has made it possible to rationally design the next generation of programmable, adaptive and patient-specific biomaterials, opening up new possibilities for precision medicine and regenerative medicine.

## 3 Transcriptome-level regulation of material-cell interactions

### 3.1 Single-cell transcriptomics

Single cell transcriptomics (scRNA-seq) is a technology that analyzes gene expression at the single cell level, which can reveal in high resolution how biomaterials affect the fate of cells. Unlike tissue sequencing, scRNA-seq does not regard tissue as a uniform whole, but reveals the differences and heterogeneity between cells [17]. This provides important information for understanding the diversity and dynamic state of complex tissues (such as bones and immune microenvironments), in which different cell groups play a key role in development, repair and immune regulation [18]. scRNA-seq aims to study the changes in gene expression under different conditions or disease states. Differential expression analysis measures the changes in transcript abundance, while cluster analysis measures co-regulating gene grouping. These methods together reveal the molecular mechanism and biological pathways [19,20].

**Fig. 2:**
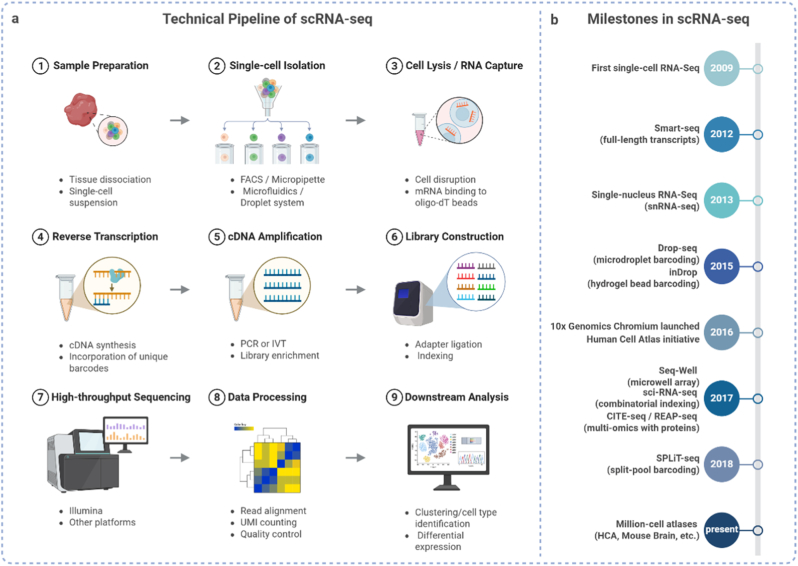
**scRNA-seq workflow and timeline.** (a) The main technical steps of scRNA-seq. (b) Key milestones in scRNA-seq development.

scRNA-seq includes several key steps: separation of cells or nuclei, droplet-based barcode marking, library construction, high-throughput sequencing and computational analysis, which are usually combined with spatial information integration. Among the high-throughput droplet sequencing platforms, the most widely used are 10x Genomics Chromium, DropSeq and inDrop, which use microfluid control technology to capture individual cells through microbeads with unique barcodes. These systems can achieve large-scale parallel sequencing and provide powerful tools for the study of transcription reactions caused by biomaterials [21,22] (Fig. 2).

The maturity of scRNA-seq is reflected in its broad application across immunology, oncology and developmental biology, which also demonstrates its potential in materiobiology [23]. For example, Villani et al. used scRNA-seq to redefine the composition of human blood dendritic cells and monocytes. Researchers have discovered a new dendritic cell subtype (AS DCs), which has a strong T cell activation ability. They also revealed the diversity of CD1C + dendroid cells and confirmed the existence of conventional dendroid cells in the blood. These findings not only improve the classification system of immune cells, but also provide new potential targets for immune monitoring and treatment [24]. Similarly, Gaiti et al. combined scRNA-seq with DNA methylation analysis to study the mechanism of Ibutinib in chronic lymphocytic leukemia. By analyzing the changes in methylation, they constructed a cell lineage tree and marked the transcription status in it. The results show that Ibutinib does not have the same effect on all leukemia cells, but selectively affects some subclones. In these subgroups, the genes associated with the cell cycle and Toll-like receptor signals were significantly up-regulted. This shows that drug treatment can not only inhibit the progression of the disease, but also reshape the lineal changes and gene expression patterns of leukemia cells [25]. In the study of developmental biology, Cao and others drew a large-scale single-cell map covering the process of organogenesis in mice. They analyzed about two million cells in the 9.5- to 13.5-day embryonic period, identified 38 major cell types and hundreds of subtypes, reconstructed the developmental trajectory, found many new marker genes, and provided a detailed picture of the molecular mechanism of organ formation [26]. In addition, the Human Cell Atlas program further demonstrates the maturity of this research method, which aims to depict all human cell types with a single cell resolution [27]. These studies show that scRNA-seq technology is both reliable and has a wide application value. It can not only reveal new laws of organ development and disease mechanisms, but also provide an important reference for biomaterial design and organ-like research.

The interaction between biomaterials and cells usually shows significant spatial and time heterogeneity. scRNA-seq enables researchers to track how different cell subgroups perceive and respond to the properties of materials, such as stiffness, porosity and surface chemical properties. It can also reveal whether biomaterials promote or inhibit line-specific differentiation, including ossification, cartilage or immune cell polarization. These findings help to reveal the molecular mechanism of material-cell interaction and provide evidence-based feedback for material design, so as to optimize the performance of biomaterials and promote accurate research in this field. Cherry et al. used computational methods to analyze the local tissue microenvironment after the implantation of biomaterials. They developed a program called Domino, which reconstructs the ligand-receptor-transcription factor network inside and outside the cell from the transcription factor activation signal. Research shows that different materials induce different immuno-matrix interactions, which directly determine the results of repair or fibrosis. In IL-17 receptor knockout mice, researchers further confirmed this mechanism and found a significant reduction in PCL-related fibrosis signals [28]. In another study, Huang et al. used scRNA-seq to analyze the cell community around the implanted silk stent. Their analysis revealed the existence of a variety of macrophage subgroups with different functions. These immune cells were identified as the major contributors to scaffold degradation and the progressive decline in mechanical stability. Further analysis suggested that scaffold structure may regulate macrophages through the Itgav-MAPK1-Stat3 signaling pathway. This study revealed the cellular and molecular basis of biomaterial degradation and highlighted that scaffold design should not only focus on initial mechanical properties but also consider its regulation of specific immune cell subpopulations [29].

### 3.2 Transcriptomics

Transcriptomics refers to the study of all RNA transcripts expressed in cells or tissues under specific conditions. It provides a global view of gene activity, revealing which genes are activated or inhibited by environmental signals, diseases or material interventions. In the field of materiobiology, transcriptomes help to reveal how biomaterials regulate cell procedures at the system level, which is a supplement to the fine resolution provided by the single-cell method. Technological progress has promoted the development of transcriptomics from hybridization-based microarrays to sequencing-based platforms. Microarrays have achieved the first large-scale gene expression survey, but their dependence on pre-sequence information and cross-hybridization problems limit the discovery of new genes. RNA-Seq overcomes these obstacles by directly reading cDNA fragments with high sensitivity and wide dynamic range. This can not only accurately quantify the transcript level, but also detect new isomers, non-coding RNA and variable splicing events [30,31]. Library construction strategies include poly(A) selection or rRNA removal, followed by high-throughput sequencing and computational analysis. In recent years, advances such as long-reading long-term sequencing and spatial transcriptometics have further expanded the ability to analyze the structure of transcripts in tissues and their spatial background [32].

Transcriptomes has been widely used in biomedical research. In the fields of immunology and autoimmune, it has identified molecular characteristics related to interferon, IL-1 and IL-17 pathways, thus guiding the development of targeted therapy [33]. In the field of oncology, it reveals the regulatory role of long-chain non-coding RNA in tumor progression [34]. In the field of toxicology, transcriptome analysis can capture changes in early gene expression after exposure to exogenous substances, thus providing in-depth insights into the study of the mechanism of hepatotoxicity and pulmonary toxicity [35]. These examples show how transcriptionomes reveals system-level responses and guides transformation strategies.

In the field of materiobiology, transcriptomics can provide important information about how cells and tissues respond to biomaterials as a whole. It can identify whether the stent induces osteogenic-related genes, inhibits pro-inflammatory cytokines or enhances angiogenic pathways. In particular, transcriptomics has been widely used to compare different biological material designs and reveal their molecular effects. Guerrero et al. designed two bone repair stents based on tricalcium phosphate (TCP) and analyzed them using transcriptomics and other tomology methods. The results show that only fine fiber stents can promote angiogenesis and ossation. This study reveals the differences in overall gene expression induced by different scaffold structures, and provides molecular evidence of how materials regulate immune response, angiogenesis and bone formation during repair [36]. Similarly, transcriptomics is also used to evaluate the response of cells to nanomaterials. Murali and others used transcriptomics to analyze human bone marrow mesenchymal stem cells (hMSCs) treated with different inorganic nanomaterials. They found that different materials induce different gene expression patterns in cells. Among them, nanosilicates were able to stabilize the skeletal progenitor state of stem cells, upregulate genes related to endochondral ossification, and finally enhance mineralized matrix deposition [37]. These studies highlight the value of transcriptomics in revealing how material composition and structure shape cellular function. By linking transcriptome changes to material properties such as stiffness, porosity or surface chemistry, researchers can better understand material - cell interactions and design more effective biomaterials. Importantly, transcriptomics provides a global background against which single-cell data can be interpreted, making it a valuable component of multi-omics strategies (Table 1).

**Table 1:** Applications of transcriptomics in materiobiology and related biomedical research.

Application area	Key findings	Contributions	Ref.
Technological advances	Transition from microarrays to RNA-Seq	Improved sensitivity, dynamic range and detection of novel isoforms and ncRNAs	[38]
Immunology	Identification of interferon, IL-1 and IL-17 gene signatures	Guidance for targeted immunotherapies	[39]
Toxicology	Early detection of xenobiotic-induced hepatotoxicity and pulmonary toxicity	Biomarker discovery	[40]
Oncology	Long non-coding RNAs regulate proliferation, survival, migration	Linked to cancer progression	[41,42]
Spatial transcriptomics	Resolves gene expression in tissue architecture	Linking molecular states to spatial context	[43]
Materiobiology	Reveals how biomaterials regulate osteogenesis, angiogenesis and immune polarization at system level	Complements single-cell approaches	[44]

### 3.3 RNomics

RNomics focuses on identifying and analyzing non-coding RNAs (ncRNAs), with particular emphasis on small RNAs that do not encode proteins but regulate RNA metabolism and post-transcriptional processes. Unlike transcriptomics, which mainly quantifies messenger RNAs, RNomics uncovers regulatory RNA species such as small nucleolar RNAs (snoRNAs), microRNAs (miRNAs) and small interfering RNAs (siRNAs). These molecules guide RNA modifications, control messenger RNA (mRNA) stability or fine-tune translation, thereby exerting broad influence over gene expression networks. In recent years, experimental and computational RNomics have uncovered many new RNA classes, including orphan snoRNAs with unknown targets and tissue-specific expression patterns, as well as miRNAs and siRNAs that control developmental programs or antiviral responses. Studies have found that some snoRNA can also act directly on mRNA, which makes the process of RNA regulation more complex than originally thought. These findings demonstrate that ncRNAs are not passive byproducts but dynamic regulators that act as molecular switches [45].

Experimental RNomics begins with total RNA extraction followed by size selection of small RNAs. Adaptor ligation, reverse transcription and small-RNA cDNA library construction enable random cloning and sequencing of expressed species. Expression is then verified by Northern blot, and function is refined using 5′/3′ RACE, transcription start site mapping, and rifampicin run-out half-life assays. These steps resolve whether candidates arise from independent transcription units or from processing of mRNA leaders/trailers and help quantify RNA stability. In parallel, computational RNomics screens intergenic regions using conservation, RNA secondary structure prediction, and machine-learning/comparative genomics features; crucially, all predictions require experimental confirmation and functional assignment. In bacteria, this combined strategy established that sRNAs can be independently transcribed or processed, and that some act within RNA - RNA interaction circuits.

In mammalian and model systems, experimental RNomics from mouse brain libraries identified 201 small non-messenger RNA candidates, including extensive C/D and H/ACA box snoRNAs that guide rRNA/snRNA modifications; several snoRNAs showed brain-specific expression, highlighting tissue-restricted ncRNA programs. These findings framed the modern view of snoRNA architecture and gene organization in relation to RNA modification. In *E. coli*, a shotgun RNomics atlas expanded the validated sRNA repertoire to 62 species, uncovered parallel transcriptional output (sRNAs co-expressed with mRNAs via processing), documented sRNA-sRNA interaction (RyeB with SraC/RyeA) requiring RNase III cleavage, overturning the assumption that sRNAs are uniformly stable. Moreover, two sRNAs (SroA/SroG) embedded riboswitch elements (THI/RFN), linking metabolite sensing to sRNA function. Clinically, RNomics has informed diagnostics: the npcTB_6715 sRNA gene enabled a multiplex PCR assay for *Mycobacterium tuberculosis* with 98 % sensitivity and 96 % specificity in 500 clinical cultures, illustrating the translational potential of sRNA gene targets. In cardiovascular diseases, the level of miR-2909 in peripheral blood mononuclear cells is closely related to the severity of coronary artery blockage, and also affects inflammatory reactions and lipid metabolism pathways. This shows that the expression characteristics of non-coding RNA can reflect the active state of the disease [46].

In material biology, ncRNA can not only act as a sensitive marker of cell response to materials, but also actively regulate cell behavior caused by materials. Specific miRNA and snoRNA patterns can reflect whether the stent is conducive to osteogenesis or cartilage differentiation, promotes vascular growth, or guides immune cell polarization or inflammatory response. RNA heology further provides insight into the mechanism, because interventions such as RNA interference or antisense strategies can be used to verify whether certain material properties work through specific ncRNA pathways, thus strengthening the design-test-improvement cycle. In cases of concern about infection, the diagnosis method based on small RNA provides an example of how to include RNA markers in the biomaterial evaluation to detect microbial contamination or harmful inflammatory reactions [47].

## 4 Genome to proteome: Structural and functional foundations of materiobiology

### 4.1 Genomics

Genomics is the comprehensive study of the complete DNA sequence of living organisms and has become the cornerstone of the omics era. Since the Human Genome Project, the rapid development of sequencing technology - from Sanger sequencing to next-generation and third-generation sequencing platforms - has enabled large-scale genetic variation analysis to be conducted with unprecedented resolution [48]. These advances are changing the way of biomedical research and having an increasing impact on materiobiology. Through genetic information, researchers can better understand how organisms react to biomaterials. Specifically, genomics reveals how different genetic mutations affect processes such as bone formation, immunomodulation and tissue repair, which are closely related to the interaction of biomaterials. Whole genome and exon sequencing can detect mutations related to rare bone diseases, thus providing potential molecular targets for new biomaterial treatment schemes. Genome association research can also find common genetic polymorphisms that affect bone density or implant integration, providing a basis for personalized material design. In addition, macrogenome sequencing can be used to analyze the microbiota at the interface of materials and tissues, thus helping to identify the risk of implant infection at an early stage. The combination of these research methods makes genomics an important basis for understanding the interaction between materials and hosts, and also provides new ideas for the rational design of a new generation of biomaterials.

Modern genomic research mainly includes whole genome sequencing (WGS), whole exon sequencing (WES), whole genome association analysis (GWAS) and macrogenome sequencing. WGS and WES can identify functional variations in the host genome and link them to material-related diseases. For example, exon sequencing of patients with rare bone diseases found that mutations in the COL1A1 and COL1A2 genes were the main cause of osteogenic insufficiency. These mutations will disrupt the synthesis of collagen and the formation of bone matrix, providing precise molecular targets for the design of biomaterials that can simulate or replace defective structures [49]. GWAS analyzes genotype–phenotype associations in populations and can identify loci that affect bone repair and implant integration. Large studies have shown that WNT16 and RSPO3 are strongly associated with bone mineral density. Since bone density directly affects scaffold fixation and the long-term stability of implants, genetic markers identified through genomic studies can serve as predictors of implant risk and provide guidance for selecting patient-specific materials. Metagenomic sequencing (mNGS) provides an unbiased profile of microbial communities and is important for infection monitoring. In clinical cases of joint replacement, mNGS has identified pathogens such as *Staphylococcus aureus* and Streptococcus even when traditional culture was negative. This method supports early infection diagnosis and the development of anti-infective biomaterial coatings [50].

**Fig. 3:**
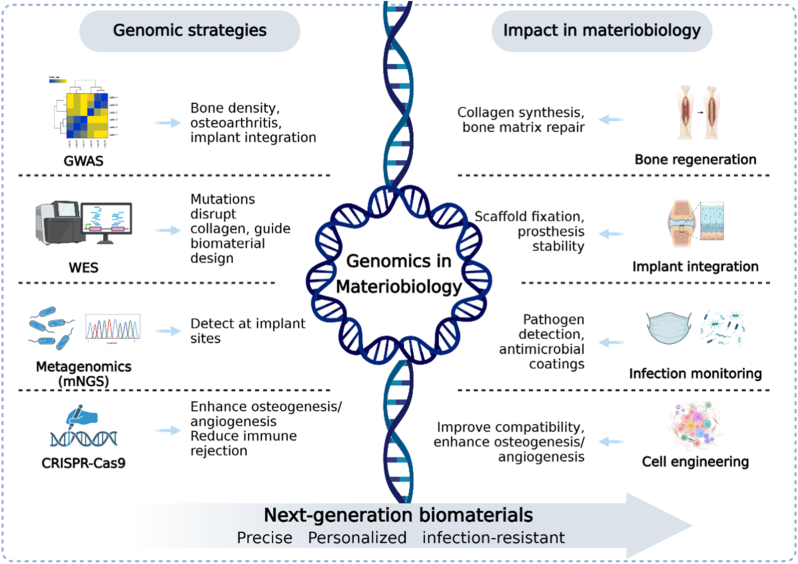
Genomics in materiobiology.

In materiobiology, the value of genomics is becoming increasingly evident. Whole-genome and exome sequencing have revealed how genetic background fundamentally influences tissue repair, as shown in patients with osteogenesis imperfecta where COL1A1 and COL1A2 mutations disrupt collagen synthesis and bone matrix formation, providing molecular targets for biomaterials designed to mimic or replace defective collagen. The whole genome correlation study further enriched the understanding of this aspect, and found gene sites closely related to bone density and osteoarthritis, such as WNT16, RSPO3 and SOST. These pathways directly affect scaffold or prosthesis integration and offer a genetic basis for personalized material selection. At the same time, mNGS has enabled rapid detection of implant-related infections, identifying pathogens such as *Staphylococcus aureus*, Streptococcus, and rare microbes even when conventional culture results are negative, thus guiding infection control and the design of antimicrobial coatings. More recently, CRISPR-Cas9 gene editing has opened new opportunities to engineer cells with specific traits, such as enhancing osteogenic or angiogenic pathways or reducing immune rejection, thereby improving the compatibility of cells with biomaterials. Together, these advances show that genomics not only reveals the genetic basis of host–material interactions but also provides powerful strategies for the precise design and optimization of next-generation biomaterials [51] (Fig. 3).

### 4.2 Proteomics

Proteomics is the systematic and large-scale study of the entire protein repertoire of cells or organisms, and it is an important part of omics research in materiobiology. Proteins are the main effectors of cellular functions. They perform key tasks such as metabolism, signal transduction, and structural support, and they translate genetic information and environmental cues into measurable physiological outcomes. Unlike the relative stability of the genome or the expression profile of the transcriptome, the proteome is highly dynamic. It is regulated by alternative splicing, post-translational modifications (such as phosphorylation, acetylation and glycosylation), protein-protein interactions, and spatial and temporal control. This complexity means that the functional state of cells in a specific microenvironment cannot be fully explained by genome or transcriptome data alone. In contrast, proteomics can directly reflect the changes in signaling pathways and effect proteins. In material biology, proteomics is very important because it can help us understand how cells perceive the physical and chemical properties of materials. At the same time, it can also reveal how cells integrate these signals and regulate processes such as differentiation, immune response and tissue regeneration [52,53].

In the commonly used “btom-up” analysis, researchers will first decompose the protein into shorter peptide segments, then separate it by high-performance liquid chromatography (LC), and finally detect it with tandem mass spectrometry (MS). If combined with multidimensional LC (such as strong cation exchange and reverse phase LC), the complexity of the sample can be further reduced, so as to obtain more comprehensive analysis results. Compared with this, “top-down” analysis directly studies complete proteins. This method can more accurately observe different post-translational modification forms, but the experimental operation is more complex and the technical requirements are higher [54]. Quantification can be achieved either label-free, by comparing spectral intensities or counts, or through stable isotope labeling methods such as SILAC, tandem mass tags (TMT), and iTRAQ, which allow multiplexed comparison of samples. More recent developments, including data-independent acquisition (DIA) and SWATH-MS, provide deeper coverage and improved reproducibility by systematically fragmenting all peptides within defined mass windows [52]. Furthermore, specialized workflows such as phosphoproteomics, glycoproteomics, and acetylomics allow researchers to follow dynamic signaling pathways and extracellular matrix (ECM) remodeling in real time, which are particularly relevant in tissue repair and immune responses [53].

MS-based proteomics has been applied to several key areas. The analysis of protein adsorption on biomaterial surfaces: LC-MS/MS studies showed that titanium implants with different surface roughness attract distinct sets of plasma proteins, which in turn regulate integrin-mediated adhesion and focal adhesion kinase signaling in osteoblasts [55,56]. Such surface-dependent protein was directly linked to differences in early bone - implant integration. In bone regeneration, proteomic profiling of cells cultured on hydroxyapatite and collagen scaffolds revealed upregulation of osteogenic proteins including type I collagen, osteocalcin and bone morphogenetic proteins (BMPs), together with increased TGF-β signaling, explaining the scaffolds’ osteoinductive effects [57]. Similarly, proteomic analysis of macrophages seeded onto degradable polymer scaffolds uncovered shifts in cytokine-related proteins and phosphorylation of NF-κB regulators, identifying the molecular switches that control M1/M2 polarization in response to material chemistry. Beyond regeneration, dendritic cell proteomics in contact with synthetic hydrogels has identified changes in antigen-processing proteins and co-stimulatory molecules, shedding light on how biomaterials may shape adaptive immune responses [[58], [59], [60], [61]]. Guerette et al. used quantitative proteomics and found that different biomaterials drive cells to produce distinct protein expression profiles. These changes mainly involve key pathways such as signal transduction, ECM remodeling, and inflammation regulation. They also identified several candidate proteins that can serve as markers of biomaterial effects and help predict how materials influence cell function and regeneration [62]. Together, these examples show how proteomics provides molecular-level evidence of cell - material interactions, guiding the rational design of scaffolds and implants with tailored regenerative or immunomodulatory functions.

## 5 Metabolomics and lipidomics: Energy flow and membrane dynamics in material responses

### 5.1 Metabolomics

Metabolomics is the systematic and comprehensive study of low-molecular-weight metabolites, including amino acids, lipids, sugars, nucleotides and other intermediates of biochemical pathways. These metabolites represent the final products of cellular regulatory processes and therefore provide the closest link between molecular mechanisms and observable phenotypes. Unlike the genome, which reflects genetic potential, or the transcriptome and proteome, which indicate intermediate layers of regulation, the metabolome captures the integrated outcome of gene expression, protein activity, enzymatic regulation, nutrient supply, and environmental exposure. One of the defining features of metabolomics is its sensitivity to dynamic changes. Because metabolite levels can fluctuate within seconds to minutes in response to stimuli, metabolomics offers a real-time window into cellular physiology. Metabolomics is very useful in the study of cell processes, such as energy generation, redox regulation, amino acid metabolism and lipid metabolism. Metabolites are not only the final product of biochemical reactions, but also play the role of “messengers” in the signaling pathway. For example, lactic acid can regulate the activity of immune cells, while succinic acid helps stabilize hypoxia-inducing factors, indicating that they are both biomarkers and important factors in regulating cell behavior [63,64]. In terms of research methods, metabolomics mainly relies on high-resolution technologies, such as nuclear magnetic resonance (NMR) and MS. These techniques are usually used in combination with separation methods, such as LC, gas chromatography (GC) or capillary electrophoresis (CE), which can not only carry out comprehensive non-targeted analysis, but also accurately detect specific metabolites. In recent years, the emergence of technologies such as stable isotope tracer and metabolic flow analysis has enabled researchers to track the dynamic changes of metabolic pathways in real time, not just measure static levels. At the same time, the development of new technologies such as spatial metabolomics and single cell metabolomics also allows us to observe metabolic differences at the level of tissue and microenvironment. It is because of these advances that metabolomics has become an important foundation of modern system biology. It can not only provide biomarkers for disease diagnosis and prognosis, but also reveal the intrinsic connection between molecular regulation and physiological function. With these characteristics, metabolomics has gradually become a key link in materiobiology, because cells play a central role in metabolic reprogramming when dealing with biomaterials [[65], [66], [67]].

**Fig. 4:**
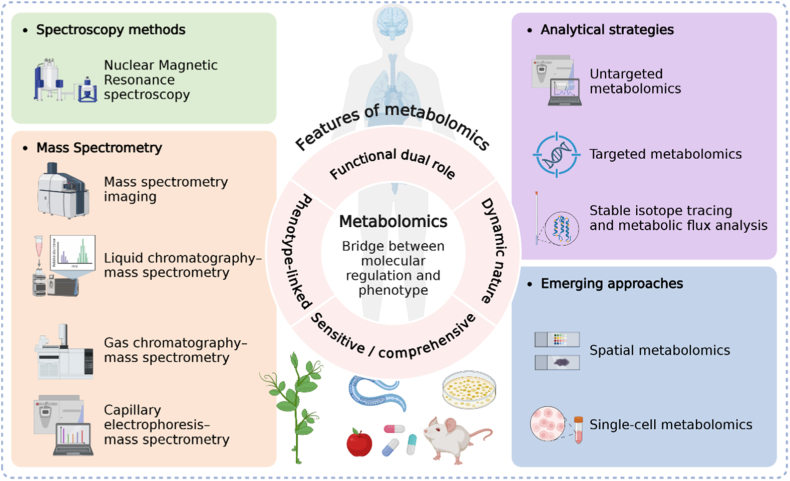
Metabolomics promotes the development of materiobiology.

Metabolomics provides us with a new perspective to help us understand how biomaterials affect cell behavior. For example, when mesenchymal stem cells are cultured on different hardness substrates, different metabolic characteristics will appear: soft matrix is more conducive to glycolysis and cartilage differentiation, while harder matrix promotes oxidative phosphorylation and osteogenic differentiation. The integration of proteomics-metabolomics further reveals that the scaffolds that release biologically active ions can promote the upregulation of tricarboxylic acid cyclic intermediates and glutamine metabolism, thus enhancing bone formation. Immunometabolism studies show that macrophages exposed to degradable polymer scaffolds undergo arginine metabolic transformation, which determines the polarization of macrophages to M1 or M2, thus linking the chemical properties of the material with immune results [64]. In addition, metagenomic - metabolomic approaches demonstrated that gut microbial metabolites such as short-chain fatty acids can influence systemic immune tone and even the success of implant integration. Representative applications highlight the translational potential of metabolomics [68] (Fig. 4).

In oncology, metabolic profiling has revealed reprogramming of glucose and amino acid metabolism, leading to diagnostic and therapeutic strategies [69]. Similar concepts can be applied to biomaterials research, where shifts in central carbon metabolism act as markers of osteogenic or angiogenic potential. Spatial metabolomics approaches, originally developed in plant biology, are being adapted to study metabolic landscapes at scaffold - tissue interfaces [67]. Moreover, lessons from reproductive medicine - where metabolomics of follicular fluid or embryo culture medium predicts developmental competence - can inform quality control of organoid cultures and biomaterial-based tissue constructs [70].

### 5.2 Lipidomics

Lipidomics is the comprehensive and large-scale analysis of the full spectrum of lipid molecules within cells, tissues, or organisms. Lipids constitute one of the most diverse biomolecular classes, encompassing phospholipids, sphingolipids, glycerolipids, sterols, and many derivatives with distinct structural and signaling functions. Beyond their fundamental roles as structural elements of membranes and reservoirs of metabolic energy, lipids serve as critical regulators of cell signaling, apoptosis, membrane trafficking, and intercellular communication. For example, eicosanoids derived from arachidonic acid regulate inflammation, while sphingosine-1-phosphate controls angiogenesis and immune cell migration. Lipids are both structural components and bioactive mediators, which make them at the intersection of metabolism and signal transduction, and endow lipomics with a unique position relative to genomics, transcriptomics or proteomics. Importantly, lipid metabolism is closely related to membrane dynamics, lipid raft tissue and receptor aggregation, all of which directly affect the immune response and tissue homeostasis. From a technical point of view, lipomics mainly relies on high-resolution MS, which provides the sensitivity and specificity required for the analysis of thousands of lipids in different categories. Shotgun lipomics can be directly injected into lipid extracts for rapid global analysis, while LC-MS and gas chromatography-mass spectrometry (GC-MS) can be used to separate isomers or low abundance lipids. Capillary electrophoresis mass spectrometry (CE-MS) and ion mobility spectrometry mass spectrometry (IM-MS) have further improved the structural resolution and can distinguish lipids with slight differences in chain length, saturation or stereochemical properties. Mass spectrometry imaging (MSI) can directly map the spatial distribution of lipids in tissues, so as to further understand how lipid heterogeneity affects the local microenvironment. In recent years, significant progress has been made in single-cell lipomics and spatial lipomics. Single cell lipomics can characterize the lipid diversity and metabolic status at the single cell level; Space lipomics combines MS with advanced imaging technology to analyze the lipid network at the tissue level. These techniques can not only quantitatively analyze the abundance of lipids, but also track the changes of lipid composition, modification and localization, so as to link lipid dynamics with physiological and pathological processes.

In the fields of biology and medicine, lipid tomics has been confirmed that lipid remodeling plays a key role in a variety of physiological and pathological processes. For example, platelet lipidomic research found that after platelets are activated, membrane phospholipids are rapidly remodeled and produce powerful lipid mediators, such as thrombin A2 and prostaglandin E2. These biologically active molecules play a central role in platelet aggregation, hemostasis and thrombosis, and their disorder is closely related to the risk of cardiovascular disease [71]. In the field of oncology, lipidomic analysis continues to find abnormal patterns of phosphatidylcholine, phosphatidylethanolamine and sheath lipids in tumor tissue. These changes promote the enhancement of membrane biosynthesis, thus accelerating cell proliferation, driving epithelial-interstitial transformation (EMT) and migration, and regulating immune escape by secreting immunosuppressive arachitic acid. These findings support the use of specific lipid species as diagnostic and prognostic biomarkers for a variety of cancers, and even as therapeutic targets [72]. In the field of neuroscience, lipotomics revealed significant changes in the brain tissue and plasma of patients with Alzheimer's disease and the derivatives of sheath lipids, ceramides and cholesterol. These changes will destroy the transport of synaptic vesicles, impair the survival of neurons, and be associated with cognitive decline, thus opening up a new path for therapeutic interventions based on lipid metabolism regulation [73].

After the implantation of biomaterials, the earliest cellular reactions usually involve the remodeling of the plasmic membrane and the production of lipid media, which initiate downstream signal cascading reactions. Lipidomics can reveal how scaffold characteristics (such as surface chemical properties, stiffness and nanomorphology) affect lipid raft tissue, integrin aggregation and adhesion spot assembly. For example, titanium implants with nanoscale roughness have been shown to induce unique fat raft-related characteristics, thereby promoting the adhesion and differentiation of osteoblasts. In the process of bone regeneration, the metabolism of phospholipids and sheath lipids is closely related to osteogenesis and angiogenesis. The increase in the levels of hemolytic phospholipid and sheath alcohol-1-phosphate in the stent culture is related to the enhancement of vascular growth and mineralization, which provides mechanical evidence for the lipid-mediated regeneration process. In terms of immunomodulation, lipomics can analyze the polarization of macrophages and dendritic cell activation induced by biomaterials. For example, the transformation of arachidonic acid metabolism to prostaglandins is conducive to the formation of pro-inflammatory M1 phenotypes, while the enrichment of disinsates and protectors derived from omega-3 fatty acids supports anti-inflammatory M2 polarization. Similarly, the changes in sheath lipid metabolism in dendritic cells are related to changes in antigen presentation and co-stimulation signals, thus linking material properties with adaptive immunoregulation.

## 6 Glycomics and immunomics: Decoding immune-material interfaces

### 6.1 Glycomics

Glycomics is a systematic study of all glycans in cells, tissues and organisms, and has become one of the core disciplines in the era of omics. Unlike nucleic acids and proteins, glycans' biosynthesis is not template driven, but is regulated by the complex glycosyltransferases, glycosidases and substrate networks in the Golgi apparatus and endoplasmic reticulum. This nonlinear and environment-dependent biosynthesis produces significant structural heterogeneity, ranging from simple monosaccharide modification to highly branched n-and O-linked glycans, glycosaminoglycans and glycolipids. This diversity endows glycans with important biological functions, including regulating protein folding and stability, regulating receptor aggregation and cell adhesion, controlling innate and adaptive immune responses, and mediating host pathogen interactions. It is worth noting that changes in the structure of sugar chains often reflect the physiological state more sensitively than changes in genes or proteins. Therefore, glycomics is considered an important supplement to genomics, transcriptomics and proteomics, and is also an indispensable part of the study of life activities [74].

High-resolution MS has become a core tool in this field, especially when it is combined with separation technologies such as LC, CE or matrix-assisted laser desorption ionionation time-of-of-flight mass spectrometry (MALDI-TOF) [75]. Quantitative methods established by strategies such as isotope labeling, metabolic precontors or fluorescent labeling can sensitively detect the content of sugar chains and their subtle structural changes. The application of new technologies such as data non-dependent acquisition (DIA) has further improved the repeatability and detection depth of the experiment. In addition, the emerging “scattering” glycomic method can quickly analyze the overall sugar chain spectrum, while the sugar chain chip technology can screen the interaction between sugar chains and proteins with high throughput, providing an important tool for the study of immunoidentification and pathogen binding mechanisms. Recent progress in spatial glycomics and single-cell glycomic approaches has added another dimension by resolving cell type - specific and tissue-contextual glycosylation patterns [76]. The parallel integration with nanotechnology has further expanded its scope of application: nanoparticle-assisted sugar capture and imaging improve detection sensitivity, and sugar-functionalized nanocarriers are being used for real-time monitoring of targeted drug delivery and sugar-mediated processes. In short, these innovations make glycomics a powerful analytical and mechanism research tool that can reveal the complexity of sugar biology and its impact on health, disease and the interaction of biomaterials [77].

The biological significance of glycomics has been fully confirmed in disease research. Age-dependent changes in IgG N-glycosylation, especially the loss of galactose and salivary acidification, have been identified as reliable biomarkers for biological aging and longevity, and are associated with systemic inflammation and immune aging [78]. In oncology, abnormal glycosylation of cell surface proteins (such as integrin, calcium mucin and mucin protein) can promote tumor progression by enhancing the potential of cell adhesion, migration and metastasis. Salivary acidification and elevated levels of rock algae glycosyl glycan can promote immune escape by binding to inhibitory Siglec receptors on immune cells, thus weakening anti-tumor immunity. In liver disease, clinical research using GlycoCirrhoTest has successfully predicted the occurrence of hepatocellular carcinoma in patients with cirrhosis, which shows higher sensitivity and specificity than traditional biomarkers such as alpha-fetoprotein (AFP) [79]. Viral pathogens further highlight the functional correlation of glycosylation: HIV-1 Env protein is highly glycosylized, forming a “sugar shield”, protecting the viral epitope from the attack of neutralizing antibodies; and the glycosylation of influenza hemoglutin changes dynamically, thus regulating receptor binding and immunoidentification, providing vaccine research and development. Key information [80].

Glycomics has a unique value in clarifying how cells perceive and respond to biomaterials. The sugar chain on the components of cell surface receptors, adhesion molecules and ECM is the primary mediator recognized by the cell after contact with the implanted material. Analyzing the interaction between sugar chains and materials can reveal how the chemical properties, morphology or degradation products of the scaffold affect cell adhesion, integrin aggregation and downstream signal conduction. Take glycosaminoglycans (GAGs) as an example, molecules such as hyaluronic acid and chondroitin sulfate not only play a structural support role in tissue engineering stents, but also regulate the osteogenic differentiation and angiogenic signal conduction of mesenchymal stem cells by binding with vascular endothelial growth factor (VEGF). Using the glycomic method, researchers can monitor the remodeling of these GAGs during tissue repair, so as to dynamically evaluate the biological properties of the scant. In addition, the results of sugar-centered analysis show that the glycosylation state of dendritic cells and macrophages will be significantly affected by the characteristics of the material, and this change will further affect the direction of their immunopolarization, making them show pro-inflammatory or repair-promoting phenotypes. For example, when the N-sugar chain branches on macrophage receptors increase, they tend to polarize to M2-like phenotypes, thus promoting tissue repair. By integrating glycomics into materiobiology, researchers can identify molecular features that link biomaterial signals with regeneration or immune outcomes, enabling rational design of glycosylated bioactive scaffolds and advancing precise regeneration strategies.

### 6.2 Immunomics

Immunocomics refers to the systematic and large-scale study of the immune system through the integration of a variety of gromic technologies. Unlike traditional immunology, which usually focuses on isolated pathways or single molecules, immunoomics integrates genomics, transcriptomics, proteomics and computational biology to construct a complete immune network map. This systematic perspective enables researchers to capture dynamic and multi-level interactions between immune cells, cytokines and signaling pathways, which cannot be fully understood by the reductionistic method.

In recent years, technological progress has accelerated the development of immunohistomology, making it a mature field. High-density immunocomic microarrays can simultaneously detect thousands of antibody-antigen interactions, thus promoting the discovery of new autoantibodies and tumor-related antigens. Large-scale epitope prediction platform, with the support of machine learning and structural modeling, can perform computer screening of peptide segments from pathogens or tumor sources to evaluate their immunogenicity. Single-cell immunoanalysis combined with scRNA sequencing, TCR and BCR sequencing can provide high-resolution maps of lymphocyte cloning diversity, differentiation status and functional procedures. At the same time, the next-generation sequencing of the TCR/BCR library provides unprecedented insights into the differences in adaptive immune memory, cross-reactivity and individual immune response. These methods jointly achieve a detailed analysis of the molecular characteristics of host-pathogen interaction, immune tolerance, and protective immunity and pathological immunity, thus expanding the application of immunology to the era of precision medicine [81,82].

Immunology provides valuable insights into the immune regulation mechanisms of a variety of diseases. In the field of oncology, autoantibody characteristics against tumor-related antigens (TAA) have been used as non-invasive biomarkers for early diagnosis and prognosis. For example, large-scale serological analysis has identified autoantibodies against p53, HER2/neu and MUC1 in breast and lung cancer, which are related to disease staging and treatment outcomes. Proteomics and serological strategies, such as antigen serological identification (SEREX) through recombinant expression clones, have revealed new immunogenic targets, some of which have entered the clinical evaluation stage as candidates for cancer vaccines [83]. In addition, immunocomics has accelerated the development of personalized cancer immunotherapy. In glioblastoma, patient-specific new antigen vaccines based on tumor exon group and transcriptome immunomic analysis can induce multifunctional CD4^+^ and CD8^+^ T cell reactions, enhance intratumor infiltration, and be associated with survival extension in early clinical trials [84].

In the field of infectious diseases, immunocomics has completely changed the design paradigm of complex pathogen vaccines. For example, in malaria prevention and control, high-throughput epitose localization and transcriptome-guided antigen discovery technology has identified new proteins on the surface of malaria parasite spores, which are currently being evaluated as next-generation candidate vaccines. In the prevention and treatment of tuberculosis, immunocomic analysis found a variety of immune dominant antigens in *Mycobacterium tuberculosis* in addition to the classic ESAT-6 and CFP-10, which provided support for the rational design of multi-epitotic subunit vaccines. These methods overcome the limitations of traditional empirical vaccine strategies, which are often difficult to effectively combat pathogens with antigen variants or immune escape mechanisms [82].

Immunocomics provides a powerful framework for analyzing how biomaterials interact with the immune system. By systematically analyzing antigen presentation, immune cell polarization and cytokine networks, immunocomics can reveal how scaffold characteristics (such as stiffness, surface chemical properties and degradation products) affect congenital and adaptive immune responses. For example, immunocomic studies show that titanium implants with different nanomorphology will induce different macrophage polarization patterns, which in turn determines the formation of downstream tissue integration or fibrotic envelopes. It has also been found that hydrogel scaffolds containing immunomodulatory functional peptides can selectively enhance the tolerance pathways of dendritic cells, thus reducing chronic inflammation. Such research results provide new ideas for the design of bioactive materials and immunomodulatory materials, and also provide more accurate strategies for tissue regeneration and implant integration. At the same time, these findings have also opened up a new research direction for how to better mobilize and utilize the immune system in regenerative medicine.

## 7 Bone organoids and omics

### 7.1 Bone organoids and applications

Bone organoids are a three-dimensional bone tissue model reconstructed *in vitro*. It can simulate the complexity of natural bone tissue in terms of cell composition, microenvironment and function. This model not only includes major cell types such as osteoblasts, chondrocytes and mesenchymal stem cells, but also introduces endothelial cells and immune cells to reproduce the synergy between multiple cells in bone tissue. With the support of biomaterial scaffolds, bone organoids can reconstruct the spatial structure and mechanical environment similar to bone matrix. More importantly, this system builds a bridge between traditional tissue engineering and group research, allowing researchers to explore the relationship between molecular mechanisms and material properties on the same platform. Compared with traditional two-dimensional cultures, bone organoids provide a more physiological system to study cell - cell and cell - matrix interactions and their regulatory mechanisms.

In basic research, bone organoids provide a new platform for exploring the molecular pathways of bone development and remodeling, and identifying key genes and signal networks involved in osteogenesis, angiogenesis and immune regulation. In terms of disease modeling, bone organoids can simulate pathological conditions such as osteogenesis, osteoporosis or osteoarthritis, which helps to discover new diagnostic markers and therapeutic targets. From the perspective of materiobiology, the combination of omics technology with bone organoids can accurately monitor how materials regulate cell heterogeneity, transcription procedure and metabolic state in the process of bone formation. In the fields of materials science and translational medicine, bone organoids can be used as functional test systems to assess how different biomaterials affect bone formation, angiogenesis and immune response, thus promoting material design from empirical methods to mechanism-based strategies [[85], [86], [87], [88]].

### 7.2 Omics-driven insights into organoid development

Recent studies have demonstrated how combining omics tools with organoid systems can reveal the molecular basis of material - cell interactions. Wang et al. synthesized a composite hydrogel bioink inspired by bone matrix, mainly composed of gelatin methacryloyl (GelMA), AlgMA and hydroxyapatite (HAP). Using digital light processing (DLP) three-dimensional bioprinting, they built large scaffolds with fine structures and loaded them with bone marrow mesenchymal stem cells (BMSCs). This composite hydrogel shows good printability and mechanical support, and has the ability of spontaneous mineralization. During long-term *in vitro* culture, the scaffold promotes osteogenic differentiation of BMSCs and deposition of mineralized matrix. After implantation, the construct gradually develops into a bone like organ with trabecular features and exhibits mechanical properties similar to cancellous bone. Transcriptome analysis further revealed that osteogenic differentiation is promoted by activating key pathways such as PI3K/Akt [15]. Zhu et al. designed a dynamic double network hydrogel composed of GelMA and DNA strand interaction. They used the hydrogel as a scaffold for three-dimensional culture *in vitro*, and found that it can significantly promote the proliferation and differentiation of osteoblasts, and support the spontaneous formation of woven osteoid organs in the long-term culture process [89]. These findings suggest that transcriptomics can provide mechanistic explanations for material induced bone tissue formation and emphasize the importance of omics integration in organoid research.

In addition to bones, omics based methods have also been applied to other organoid systems, providing valuable references for materiobiology. Reza et al. induced human pluripotent stem cells (hiPSCs) and treated them with ascorbic acid or bilirubin to construct liver organoids with partition characteristics. They used mononuclear RNA sequencing (snRNA seq) to analyze the cellular composition and spatial characteristics of organoids. Combining transcriptomics and epigenetics analysis, this study revealed the molecular mechanism of partitioning. This research provides new ideas and theoretical support for the construction of organ-like organs with complex functions [90]. Lassé et al. used kidney organs derived from human pluripotent stem cells as a model and analyzed it by multi-hromology method. They mapped the changing trends of proteome and transcriptome during the culture process, and found that ECM proteins will increase over time, while glomerular-related proteins will gradually decrease, which shows that organs have undergone dynamic changes in structure and function during maturity. By comparing these data with patient samples, researchers found that the proteome and transcriptome characteristics of organoids are very similar to those of kidney disease tissues, and some of these key molecules may also become potential disease biomarkers.

These studies emphasize that the combination of bone organoids and multi-group analysis can not only reproduce the physiological and pathological characteristics of bones, but also provide a strong framework for mechanism-based evaluation and optimization of biomaterials. However, the construction of bone organoids still faces technical and biological challenges. Current models usually have problems such as insufficient vascularization, limited immune and neural integration, and inaccurate mineralization patterns, which limit their ability to fully reproduce natural bone complexity. It is also very difficult to maintain mechanical stability and functional maturity in the long-term cultivation process, especially considering the load-bearing and mineralization characteristics of the skeleton [91,92]. In addition, the lack of standardized cell sources, scaffold preparation and large-scale culture schemes has led to differences in results and limited their clinical transformation. In order to overcome these limitations, researchers began to conceive the gradual development of bone organoids, from an early physiological model (1.0) to a more advanced pathological model (2.0), a structural model (3.0), a composite model (4.0) and an applied model (5.0) [85]. Each stage represents a step towards greater biological complexity and transformation potential. In the end, the combination of henomic technology and organ-like engineering can reveal the key molecular regulatory factors of bone development and guide the rational optimization of organ-like composition and structure, so as to help overcome existing obstacles. This integration lays the foundation for the next generation of material biology, in which organ-like models and hemology technologies jointly guide the rational design of biological activity and personalized materials [93,94].

## 8 Future perspectives

**Fig. 5:**
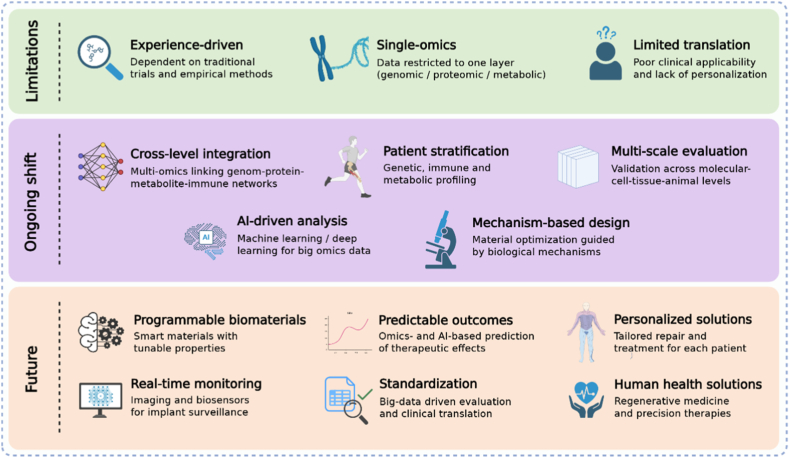
Genomics in materiobiology.

With the rapid development of multi-grouping, material biology is shifting from observation-based and experience-based research to data- and mechanism-oriented research. The future development direction is no longer limited to a single level, but integrates different group levels. Information from the genome, transcriptome, proteome, metabolome, lipidome, glycome and immune system can be integrated to build a complete map of cell interaction with matter. This integrated perspective allows scientists to track how molecular changes induced by substances affect cell fate, immune response and tissue repair(Fig. 5).

Precision medicine is expected to add new significance to materiobiology. Methods such as genomics and immunoomics make it possible to predict how differences in genetic composition, immune status and metabolic activity affect an individual's response to biomaterials. For example, the GWAS has identified the gene locations related to bone mineral density and immunomodulation, which provides guidance for the selection of bone regeneration scaffolds. Immunocomic analysis can also draw cytokine networks and T cell and B cell receptor libraries, thus supporting the development of biomaterials for immunomodulation for specific patient groups. At the same time, single-cell transcriptomics contributes to precision medicine by revealing cell diversity at the material-tissue interface, detecting rare but functionally critical immune or matrix cell subgroups, and tracking lineages or repair pathways. For example, scRNA-seq studies show that different macrophages and fibroblast groups can lead to completely opposite results after stent implantation. Some subgroups promote regeneration, while others promote fibrosis remodeling. When integrated with the data of genomics and immunoomics, these findings improve the ability to predict patients' specific responses to biomaterials at the molecular and cellular levels. In this system, the whole process from patient classification to material selection to treatment effect has become the key link to achieve precise medical treatment [95].

When AI is combined with group technology, it is becoming an important force to promote the progress of materiobiology. Modern multiomics research will produce a large amount of data, which covers information such as genetic variation, transcription dynamics, protein interaction, metabolic changes and immune characteristics. By applying machine learning and deep learning, AI can identify the underlying patterns in these data sets and link molecular signals to functions at the cellular and tissue levels. In the field of materiobiology, such analysis clarifies how material properties (including stiffness, surface chemical properties and structural tissue) affect pathways related to ossification, angiogenesis and immunomodulation. In addition to in-depth understanding at the mechanism level, the model supported by AI can also predict the performance of biomaterials by integrating the patient-specific genomic or immunocomic characteristics, so as to predict the stent integration or coating reaction in a personalized context. For example, algorithms based on large-scale genology and clinical data set training can stratify patients according to genetic background or immune status, providing practical guidance for the personalized selection of biomaterials in bone regeneration [96,97]. In addition, combining AI with imaging tools, biosensors and group data can continuously monitor implants and predict long-term efficacy, thus establishing a feedback system that connects materials engineering, bioreaction and clinical practice. By integrating genomic analysis results with AI algorithms, we can achieve purposeful design of biomaterials, as well as programmability and predictability. Moreover, it can be customized on an individualized basis according to treatment needs [98,99].

In the future, materiobiology will depend on deep interdisciplinary integration. Biology, medicine, materials science, computer science and engineering will together form a full innovation ecosystem. At the basic level, multi-omics and AI will explain cell-material interactions. At the application level, precision medicine will guide the design of personalized biomaterials. At the translational level, the combination of big data, multi-omics analysis and clinical validation will play a key role in transforming laboratory discoveries into standardized and scalable biomedical products [100,101]. Omics data from preclinical and patient studies can reveal molecular biomarkers that predict material performance, biocompatibility and long-term safety. Integrating these data sets with the patient's clinical end-point events will help to establish the evaluation criteria that can withstand deliberation, and guide us to optimize the design parameters of biomaterials based on the data. At the same time, it is very important to promote multicenter clinical trials, which can provide quantitative verification of the efficacy of materials and body reactions, and lay the foundation for the regulatory review and large-scale production [102,103]. The core purpose of the above technology path is to explore the connection mechanism and practical application. In this process, AI and machine learning technology are introduced to process omics data, which can effectively identify the relevance missed by conventional statistical methods, and build prediction models for auxiliary diagnosis or prognosis. This will form a continuous feedback mechanism: the results of calculation and prediction need to be tested by experiments, and the data of clinical feedback will in turn optimize the calculation model. In parallel, there are digital health technologies (such as mobile medical platforms and wearable sensor devices), which can collect real-world data that cannot be obtained in the traditional laboratory environment, creating conditions for evaluating the interaction between materials and the body in a more natural state. By systematically integrating these technologies and data, materiobiology is expected to change from the current description-based research model to a new paradigm that can directly guide clinical practice, improve the effect of regenerative treatment and ensure the sustainability of transformation [104].

In general, with the combination of herology technology, precision medicine and AI, the study of material biology is no longer limited to the traditional idea of “replacement” or “repair”. Now, this field is moving towards a higher stage - researchers can design biomaterials with programmable and predictable functions and make personalized adjustments according to the specific needs of different patients. These progresses are expected to completely change the research direction of tissue regeneration, promote the development of more accurate, efficient and clinical treatment methods, and ultimately truly improve human health.

## CRediT authorship contribution statement

**Peiran Song:** Writing – review & editing, Writing – original draft, Conceptualization. **Yuezhou Wu:** Writing – original draft. **Chen Zhang:** Writing – review & editing, Writing – original draft. **Fengjin Zhou:** Writing – review & editing, Conceptualization. **Long Bai:** Writing – review & editing, Writing – original draft, Funding acquisition, Conceptualization. **Jiacan Su:** Supervision, Funding acquisition, Conceptualization.

## Declaration of competing interest

The authors declare no conflicts of interest.

## Data Availability

No data was used for the research described in the article.
